# Revisits After Emergency Department Discharge for Conditions with High Disposition-Decision Variability at Hospitals with High and Low Discharge Rates

**DOI:** 10.5811/westjem.2022.3.55036

**Published:** 2022-06-29

**Authors:** Avi Baehr, Angela J. Fought, Renee Y. Hsia, Jennifer L. Wiler, Adit A. Ginde

**Affiliations:** * University of Colorado School of Medicine, Department of Emergency Medicine, Aurora, Colorado; † Denver Health Medical Center, Department of Emergency Medicine, Denver, Colorado; ‡ Colorado School of Public Health, Center for Innovative Design & Analysis, Department of Biostatistics and Informatics, Aurora, Colorado; § University of California San Francisco, Department of Emergency Medicine, San Francisco, California

## Abstract

**Introduction:**

The first proposed emergency care alternative payment model seeks to reduce avoidable admissions from the emergency department (ED), but this initiative may increase risk of adverse events after discharge. Our study objective was to describe variation in ED discharge rates and determine whether higher discharge rates were associated with more ED revisits.

**Methods:**

Using all-payer inpatient and ED administrative data from the California Office of Statewide Health Planning and Development (OSHPD) 2017 database, we performed a retrospective cohort study of hospital-level ED discharge rates and ED revisits using conditions that have been previously described as having variability in discharge rates: abdominal pain; altered mental status; chest pain; chronic obstructive pulmonary disease exacerbation; skin and soft tissue infection; syncope; and urinary tract infection. We categorized hospitals into quartiles for each condition based on a covariate-adjusted discharge rate and compared the rate of ED revisits between hospitals in the highest and lowest quartiles.

**Results:**

We found a greater than 10% difference in the between-quartile median adjusted discharge rate for each condition except for abdominal pain. There was no significant association between adjusted discharge rates and ED revisits. Altered mental status had the highest revisit rate, at 34% for hospitals in the quartile with the lowest and 30% in hospitals with the highest adjusted discharge rate, although this was not statistically significant. Syncope had the lowest rate of revisits at 16% for hospitals in both the lowest and highest adjusted discharge rate quartiles.

**Conclusion:**

Our findings suggest that there may be opportunity to increase ED discharges for certain conditions without resulting in higher rates of ED revisits, which may be a surrogate for adverse events after discharge.

## INTRODUCTION

The emergency physician’s decision to admit a patient is among the most expensive and consequential decisions in healthcare. In 2017, hospital expenditures accounted for nearly a third of the United States’ $3.5 trillion in healthcare spending,^1^ with the majority of these admissions originating from the emergency department (ED).^2^ While critical illness and minor injury carry straightforward disposition decisions, other common conditions have marked interhospital variability in discharge rates.^3^, ^4^ Studies of select populations^5^–^7^ have shown a significant burden of potentially avoidable admissions. Paired with the demonstrated interhospital variability in admission rates, this suggests an opportunity to improve healthcare value by decreasing unnecessary costs associated with avoidable admissions.^8^

To address this opportunity, the American College of Emergency Physicians has proposed an alternative payment model, the Acute Unscheduled Care Model (AUCM), which targets reducing avoidable admissions for conditions with high variability in hospital-level admission rates.^9^ This model has been endorsed by the US Secretary for Health and Human Services and is under consideration for implementation by the Center for Medicare & Medicaid Innovation as well as private payers. If adopted, this model would be the first emergency care-based alternative payment model and stands to significantly alter the landscape of value-based payments for emergency care.^9^

Reducing costs is only one component of the value equation, and the AUCM pairs the incentive to reduce admissions with an emphasis on care coordination and adverse event reduction after ED discharge.^9^ Little is known, however, about how higher ED discharge rates are associated with post-ED discharge adverse events. One study in Medicare patients found that hospitals with higher ED discharge rates had a threefold increase in mortality rates after ED discharge,^10^ while another study of Medicare patients with syncope did not find an association between ED discharge rates and post-discharge adverse events.^3^ In addition to suggesting different trends in the association between ED discharge rates and adverse event rates, these studies were limited only to the Medicare population, and to our knowledge this topic has not yet been explored in a more general ED population.

Using conditions that had been previously identified as having high variability in discharge rates,^3^, ^4^ our study objective was to describe variation in ED discharge rates and determine whether higher discharge rates were associated with higher rates of ED revisits.

## METHODS

### Study Design and Setting

We performed a retrospective observational analysis of all-payer inpatient and ED administrative data from the California Office of Statewide Health Planning and Development (OSHPD) 2017 database.^11^ This database encompasses all non-federal licensed hospitals and EDs in California, which has a large, geographically and sociodemographically diverse population.^12^ We used the non-public database for our analysis, which included patient record linkage numbers that allow for tracking ED visits and admissions over time. We accessed the non-public database via an existing data request with the California Department of Health Care Access and Information, which permits nonprofit educational institutions to request and access this data for research purposes. We limited our sample to adult patients (≥18 years old) and excluded EDs with fewer than 10,000 adult patient visits in 2017 to ensure an adequate sample for condition-specific hospital-level analyses. We excluded visits with dispositions of left against medical advice, left before visit completion, other/unknown disposition, and without record linkage numbers because of the inability to precisely classify and measure outcomes for these visits.

*Population Health Research Capsule*

What do we already know about this issue?

*Interhospital variability in ED discharge rates is significant for certain conditions, and a proposed payment model may incentivize increased discharge rates.*

What was the research question?

*For conditions with interhospital variability in ED discharge rates, are higher discharge rates associated with more revisits?*

What was the major finding of the study?

*For many common conditions, EDs with higher rates of discharge were not associated with higher rates of ED revisits.*

How does this improve population health?

*Our findings suggest that there may be opportunity to increase ED discharges for certain conditions without resulting in higher rates of ED revisits, which may be a surrogate for adverse events after discharge.*

We limited our analysis to seven medical conditions that have been previously identified as having interhospital variability in admission rates: abdominal pain; altered mental status; chest pain; chronic obstructive pulmonary disease (COPD) exacerbation; skin and soft tissue infection; syncope; and urinary tract infection.^3^,^4^ We identified these conditions by primary discharge diagnosis using previously described *International Classification of Diseases 10^th^ Revision* and Clinical Classification Software codes.^3^,^4^

### Statistical Analysis

For each condition, we used logistic regression to estimate adjusted discharge rates at the hospital level, adjusting for hospital, age, gender, payer type, and Elixhauser comorbidity score.^13^ Using the adjusted mean discharge rates per hospital, we categorized hospitals into quartiles to delineate the hospitals with the highest and lowest rates of adjusted ED discharge for each condition.

Our primary outcome of interest was all-cause ED revisits within 30 days of discharge from an index visit. Index visits were defined as any ED visit for a condition of interest resulting in discharge without a visit for the same diagnosis within the previous 30 days. We limited index visits to the period from January 1–December 1, 2017 to ensure an adequate 30-day follow-up period for assessing ED revisits within the dataset. For each condition, we calculated hospital-level ED revisit rates as the number of index visits that had at least one 30-day ED revisit to any study hospital divided by the total number of index visits. Because timestamps were not available in the dataset, we did not include patients with multiple ED visits on the same day in our revisit count, since we were not able to determine whether the index visit or the other ED visit came first. We compared revisit rates after ED discharge for hospitals in quartiles 1 and 4 using bootstrap-estimated 95% confidence intervals (CI) for each condition. This study was granted human subjects approval through the University of California San Francisco Institutional Review Board.

## RESULTS

**Figure 1:**
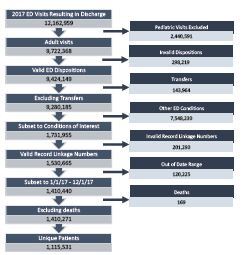
Selection of eligible index visits.

There were over 12 million visits to 271 EDs resulting in discharge in the calendar year of 2017 in the OSHPD database; the selection of our index visits can be seen in Figure 1. We excluded 3.1% of potentially eligible visits due to invalid dispositions. An additional 11.6% of potentially eligible visits were excluded due to lack of record linkage numbers. Ultimately, after exclusions and after subsetting to conditions of interest, we identified 1,410,271 visits resulting in ED discharge by 1,115,531 patients during our study period. Demographic characteristics for these visits can be seen in Table 1.

After adjusting for age, gender, payer, and comorbid conditions, we found a greater than 10% difference in the median ED discharge rates between hospitals in the highest and lowest discharge rate quartiles for every condition of interest except for abdominal pain. The spread of adjusted discharge rates was greatest for altered mental status, COPD exacerbation, and syncope, with a greater than 20% difference in the median adjusted discharge rate at the bottom and top quartiles of hospitals. For each of these conditions, hospitals in quartile 4 discharged greater than 90% of their patients, on average, while hospitals in quartile 1 had adjusted discharge rates around 70% (Table 2). Due to the lack of meaningful variability in interhospital adjusted discharge rates for abdominal pain, we excluded this condition from further analysis.

**Figure 2:**
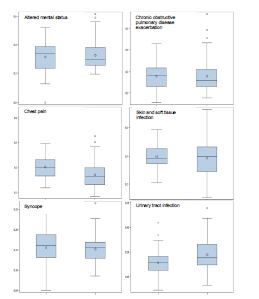
Post-discharge revisits at hospitals with high and low adjusted emergency department discharge rates. In each panel, left box plot is quartile 1 (more admissions) and right box is quartile 4 (more discharges). Y axis is ED revisit rate. ED, emergency department.

We next analyzed the differences in ED revisits and found no meaningful difference between the ED revisit rate between hospitals in quartile one, with the highest rate of admissions, and quartile four, with the highest rate of discharges. Among the six conditions with a meaningful difference in ED discharge rates, altered mental status had the highest rate of ED revisits, with a median rate of 34% (95% CI 29–37%) in hospitals with lower discharge rates and 30% (95% CI 28–33%) in hospitals with higher discharge rates. Syncope had the lowest revisit rate, with a median rate of 16% (95% CI 14–18%) in hospitals with lower discharge rates and 16% (95% CI 14–16%) in hospitals with higher discharge rates (Table 3; Figure 2).

## DISCUSSION

Our study found that while there was significant variability in ED discharge rates for the majority of conditions studied, higher ED discharge rates were not associated with higher rates of ED revisits. Our results show marked interhospital variation in ED discharge rates, even after adjusting for visit characteristics, for several common conditions; the one studied condition that did not demonstrate this variation had previously been studied in a Medicare rather than all-payer population,^3^ and we suspect this may be the reason for our difference in results. Overall, this demonstrated variability suggests an opportunity to safely reduce avoidable hospital admissions.

Consistent with previous studies,^3^,^4^ we found that common ED conditions have significant variation in ED discharge rates, and we went on to find that hospitals with higher rates of ED discharge did not have higher rates of revisits. Taken together, our findings suggest a pivotal role of the ED in serving as a gatekeeper for hospital admissions and the associated downstream costs. Prior work has found that EDs serve a critical role in readmission reduction under Medicare’s Hospital Readmissions Reduction Program.^14^ Our study shows that EDs may have an important upstream role as well in reducing avoidable admissions without placing patients at increased risk of return visits or subsequent admissions.

Reducing avoidable admissions represents a growing area of policy focus with significant implications for healthcare costs. Incentivizing higher rates of ED discharges, however, may place patients at risk for adverse events after ED discharge. While ED revisits are an imperfect marker of ED quality,^15^,^16^ they remain a source of significant expense^17^ and may represent patient dissatisfaction with clinical care or the discharge process, or lack of access to outpatient care.^18^ To our knowledge, this is the first study to analyze an association between ED discharge rates and revisits in an all-payer population.

Our finding that there was no meaningful difference in revisits after discharge between hospitals that had higher and lower adjusted rates of ED discharge has potential implications for future initiatives aimed at reducing avoidable admissions. Our results suggest that it may be possible to incentivize higher discharge rates without increasing downstream acute care utilization. Further work will be required to assess any association between higher ED discharge rates and other patient-centered outcomes, such as mortality and patient-reported health outcomes and satisfaction. Importantly, one study in Medicare patients did find a higher risk of post-ED discharge mortality for patients seen at hospitals with higher discharge rates.^10^ The impact of such initiatives on patient-centered outcomes, especially in high-risk subgroups, will be important to evaluate.

## LIMITATIONS

Although the OSHPD database is comprehensive, including nearly all ED encounters in the state of California, our study was limited by the administrative data available as well as the retrospective nature of our analysis. In addition to the limitations below, our analysis was subject to unmeasured confounding. Further, our analysis was limited to one large state, and so our results might not be generalizable to other locations. For example, relative to the United States as a whole, California has a larger Hispanic population, slightly higher poverty rates, and a slightly lower proportion of the population without health insurance.^12^

Our study was also limited to visits with valid record linkage numbers in order to allow for tracking of ED revisits. Visits that were excluded due to lack of record linkage numbers tended to represent younger patients with higher rates of Medicaid or self-pay insurance coverage and could have potentially biased our results in either direction (Supplemental Table 1). Furthermore, records for admitted patients include only the final discharge diagnosis rather than the ED diagnosis. In calculating our ED discharge rates, we used the ED discharge diagnosis for discharged patients while using hospital discharge diagnosis for admitted patients. Therefore, we are likely not capturing some patients who may have been admitted with an ED diagnosis of, for example, “abdominal pain” but were subsequently found to have a definitive diagnosis, which may be listed as their primary hospital discharge diagnosis. This may have resulted in overestimating discharge rates for the symptom-based diagnoses such as chest pain and abdominal pain.

In calculating ED revisits, we were only able to capture 30-day revisits to study hospitals, and it is possible that true revisit rates were higher than presented in our analysis if patients re-presented to EDs that were either not included in the OSHPD database or were excluded from our analysis. This may have biased our results in either direction. Additionally, our model only included adjustment for patient characteristics, and we did not control for hospital or geographic characteristics. While this is consistent with models currently in use by the Centers for Medicare & Medicaid Services,^19^ it is possible that hospital-level factors accounted for some of the variability we saw in adjusted discharge rates. Previous work has demonstrated that variability in ED admission rates does persist, however, even after adjusting for hospital-level factors.^4^,^20^

## CONCLUSION

Our study did not find a relationship between higher ED discharge rates and ED revisits, which may suggest that ED discharges may be able to be safely incentivized for certain conditions without increasing the risk of ED revisits. However, further work is needed to determine whether this pattern can be demonstrated for other conditions and for other post-discharge adverse events. It will also be essential to determine the impact of specific ED interventions, such as ED observation or case management involvement, on post-discharge adverse events. As private and public insurers continue to consider alternative payment models focused on reducing avoidable admissions, it will be critical to prospectively assess the impact on patient safety, especially in high-risk populations.

## Supplementary Information



## Figures and Tables

**Table 1:** Demographic characteristics of patients discharged from the emergency department with a condition of interest (N = 1,115,531). Missing data: gender (n = 32; 0%); race/ethnicity (n =15,144; 1%); payer (n = 390; 0%).

Characteristic	Frequency	Percentage
Age		
18–34	334,899	30%
35–64	518,717	46%
65–84	208,465	19%
85+	53,450	5%
Gender		
Female	688,913	62%
Male	426,586	38%
Race/ethnicity		
Non-Hispanic White	481,514	43%
Hispanic	361,556	32%
Non-Hispanic Black	125,626	11%
American Indian or Alaska Native	6580	1%
Asian or Pacific Islander	79,626	7%
Other	45,485	4%
Payer		
Private insurance	366,931	33%
Medicare	293,416	26%
Medicaid	376,530	34%
Self pay	60,460	5%
Other	17,804	2%

*ED*, emergency department.

**Table 2:** Emergency department adjusted discharge rates, per hospital.

Condition	Quartile 1: median adjusted discharge rate (IQR)	Quartile 4: median adjusted discharge rate (IQR)	Difference in adjusted discharge rate medians between quartiles
Abdominal pain	96% (95–97)	99% (99–99)	3%
Altered mental status	73% (68–79)	96% (94–97)	23%
COPD exacerbation	69% (64–73)	90% (89–94)	21%
Chest pain	83% (74–86)	98% (98–99)	15%
Skin/soft tissue infection	76% (73–78)	91% (90–93)	15%
Syncope	77% (71–82)	98% (97–98)	21%
Urinary tract infection	84% (82–86)	97% (96–97)	13%

*COPD*, chronic obstructive pulmonary disease; IQR, interquartile range

**Table 3:** Revisit rate after emergency department discharge for hospitals with the highest and lowest adjusted discharge rate quartiles, reporting the medians and bootstrap estimated 95% confidence intervals. Abdominal pain not included due to lack of variability in discharge rates.

	Median revisit rate (95% CI)	
Condition	Quartile 1 hospitals (Fewer discharges)	Quartile 4 hospitals (More discharges)
Altered mental status	34% (29–37%)	30% (28–33%)
COPD exacerbation	28% (26–29%)	26% (25–29%)
Chest pain	20% (18–22%)	16% (15–18%)
Skin/soft tissue infection	29% (29–30%)	30% (27–31%)
Syncope	16% (14–18%)	16% (14–16%)
Urinary tract infection	23% (22–23%)	24% (23–25%)

CI, confidence intervals; COPD, chronic obstructive pulmonary disease.
