# Supplementary material for: Revisits After Emergency Department Discharge for Conditions with High Disposition-Decision Variability at Hospitals with High and Low Discharge Rates
Source: West J Emerg Med. 2022 Jun 29;23(4):564–9. doi: 10.5811/westjem.2022.3.55036 (PMC9391021; doi:10.5811/westjem.2022.3.55036)
Supplement: Supplementary file 1 [file wjem-23-564-s001.docx]

**Supplemental Table 1: Comparison of demographic characteristics of patients discharged from the ED with a condition of interest with and without record linkage numbers (RLN)**

|  | **Valid RLN** | | **Invalid RLN** | |
| --- | --- | --- | --- | --- |
| **Characteristic** | **N** | **N (%)** | **N** | **N (%)** |
| Age Group | 1,530,665 |  | 201,290 |  |
| - 18-34 |  | 456,234 (29.81) |  | 83,656 (41.56) |
| - 35-64 |  | 724,839 (47.35) |  | 100,810 (50.08) |
| - 65-84 |  | 278,244 (18.18) |  | 14,151 (7.03) |
| - 85+ |  | 71,348 (4.66) |  | 2,673 (1.33) |
| Sex | 1,530,624 |  | 201,278 |  |
| - Female |  | 944,791 (61.73) |  | 127,931 (63.56) |
| - Male |  | 585,833 (38.27) |  | 73,347 (36.44) |
| Ethnicity | 1,504,053 |  | 194,888 |  |
| - Non-Hispanic |  | 1,005,796 (66.87) |  | 69,246 (35.53) |
| - Hispanic |  | 498,257 (33.13) |  | 125,642 (64.47) |
| Race | 1,507,530 |  | 196,149 |  |
| - American Indian or Alaska Native |  | 9,455 (0.63) |  | 474 (0.24) |
| - Asian or Pacific Islander |  | 99,583 (6.61) |  | 11,339 (5.78) |
| - Black or African American |  | 192,893 (12.80) |  | 8,761 (4.47) |
| - White |  | 913,296 (60.58) |  | 105,232 (53.65) |
| - Other Race |  | 292,303 (19.39) |  | 70,343 (35.86) |
| Payer | 1,530,161 |  | 201,122 |  |
| - Private Insurance |  | 459,694 (30.04) |  | 47,126 (23.43) |
| - Medicare |  | 409,772 (26.78) |  | 11,492 (5.71) |
| - Medicaid/Medi-Cal |  | 559,037 (36.53) |  | 108,314 (53.85) |
| - Self Pay |  | 78,081 (5.10) |  | 31,299 (15.56) |
| - Other |  | 23,577 (1.54) |  | 2,891 (1.44) |
